# Rippl and the Alzheimer’s Association: A Strategic Approach to the GUIDE Model

**DOI:** 10.1002/alz.095813

**Published:** 2025-01-09

**Authors:** Kris Engskov

**Affiliations:** ^1^ Rippl, Seattle, WA USA

## Abstract

N/A N/A N/A N/A N/A N/A N/A N/A

